# Phenological Plasticity and Bio-Physiological Impacts of *Corythucha arcuata* Under Aridity and Edge Dynamics in Southern Transylvania Oak Forests

**DOI:** 10.3390/life16060935

**Published:** 2026-06-01

**Authors:** Cristina Stancă-Moise, George Moise, Anca Șipoș, Mihaela Rotaru, Cristian Felix Blidar

**Affiliations:** 1 Department of Agricultural Sciences and Food Engineering, Faculty of Agricultural Sciences, Food Industry and Environmental Protection, “Lucian Blaga” University of Sibiu, 5-7 Ion Ratiu Street, 550003 Sibiu, Romania; anca.sipos@ulbsibiu.ro; 2 Department of Industrial Engineering and Management, Faculty of Engineering, “Lucian Blaga” University of Sibiu, 4 Emil Cioran Street, 550025 Sibiu, Romania; 3 Department of Biology, Faculty of Informatics and Sciences, University of Oradea, 1 Universităţii Street, 410087 Oradea, Romania; cblidar@uoradea.ro

**Keywords:** *Corythucha arcuata*, *Quercus petraea*, aridity index, forest edge effect, physiological stress, metabolic bait, invasive species

## Abstract

The invasive expansion of the oak lace bug (*Corythucha arcuata*) represents a major threat to European oak forests, yet the synergistic roles of climatic stressors remain poorly understood. This study investigates the phenological plasticity and adaptive thermoregulation of *C. arcuata* in the specific microclimatic conditions of the Rășinari Forest District, Romania. Monitoring across an altitudinal gradient (525–825 m) identified a complex voltinism, characterized by a highly successful second generation (G_2_) and a restricted third generation (G_3_, <12% emergence due to early frosts). By utilizing a physiological time scale (GDD), we demonstrated that G_2_ exhibits a 15% temporal compression in development duration compared to G_1_. A critical tipping point for host vulnerability was identified at a De Martonne Aridity Index (I_Ar_) value of 20. Below this threshold, oak trees underwent a linear physiological decline, with a 74.5% decrease in chlorophyll content and a 58.8% accumulation of soluble sugars. These findings support the metabolic bait hypothesis, where drought-stressed foliage becomes a high-quality nutritional resource. Furthermore, we established a critical thermal threshold of 32 °C, which triggers active vertical migration from the sun-exposed canopy to shaded interiors to avoid heat stress. Our results provide a predictive framework for sustainable forest management, identifying aridity as an injury amplifier that facilitates pest impacts under a warming climate.

## 1. Introduction

In the contemporary era of the Anthropocene, biological invasions mediated by globalization and accelerated climate change represent one of the most formidable threats to the resilience of forest ecosystems worldwide [1,2]. The oak lace bug, *Corythucha arcuata* (Say, 1832), is native to the eastern and central regions of North America, spanning from southern Canada to the Gulf of Mexico. As a polyvoltine species, it exhibits an aggregative lifestyle, often forming dense colonies on the abaxial leaf surface. This study provides a comprehensive analysis of its adaptive mechanisms in newly invaded territories of South Transylvania (Romania). Rather than merely documenting a pest survey, our data elucidates the biological synchronization of *C. arcuata* with local environmental constraints.

Originally described by Thomas Say in 1832 [3], this species belongs to a complex taxonomic group characterized by specialized herbivory on the *Quercus* genus [4,5]. While maintaining a relative ecological equilibrium in its native range [6], its introduction into European and Asian habitats has triggered unprecedented outbreaks, as documented in early taxonomic and ecological surveys [5,6]. Since its initial detection in Italy in 2000 [7,8], the pest has systematically invaded Turkey (2003) [9], Switzerland (2005) [10,11], and Iran (2011) [12]. The last decade has witnessed a dramatic surge in the Balkan Peninsula and Central Europe, with critical reports from Bulgaria [13], Hungary [14,15], Croatia [16], and Serbia [17,18], where it has caused extensive forest canopy discoloration. The invasion front has further extended into Österreich [19], Slovakia [20], and Ukraine [21,22,23], reaching as far as the European part of Russia [24,25,26,27] and mainland Iberian Peninsula [28]. In Romania, the official confirmation of its presence in 2015 [29,30,31,32,33] marked the beginning of a rapid colonization phase, with recent studies highlighting its establishment in southern and western oak stands [34,35,36,37].

It has been established that the pest primarily targets the palisade parenchyma, leading to a significant reduction in net photosynthetic rates, stomatal conductance, and overall chlorophyll content [38]. While previous studies documented the rapid spread and host range across Europe [34,35,39], the specific biochemical disruptions and physiological decline are detailed in recent high-resolution analyses [38]. The physiological disruptions induced by *C. arcuata* feeding translate into measurable growth suppression. While recent studies on oak seedlings have documented significant reductions in biomass accumulation and photosynthetic efficiency, these early-stage impacts suggest a high potential for long-term growth decline in mature stands. Specifically, Tsikas and Karanikola (2025) reported that chronic infestation correlates with altered wood properties and reduced vigor in *Quercus frainetto*, although the direct impact on tree-ring increments in pedunculate oak varies depending on local environmental stressors [40]. The distribution and abundance of the pest within the canopy are governed by light intensity and leaf structural traits [41]. This micro-environmental preference suggests that trees at forest edges or those with higher solar exposure may experience more severe physiological stress due to increased pest density.

Current research has shifted toward understanding how environmental stressors, such as drought and extreme heat, modulate the bug–host interaction [42]. Specifically, Williams et al. (2021) [43] emphasize the role of climate in pest status, while the synergistic effect of aridity on host susceptibility is further explored [44]. It is well recognized that forest trees respond to multiple environmental stresses through complex messenger-level syntheses, often involving hydraulic controls and stomatal adjustments [45]. Recent evidence suggests that under acute water stress, biochemical shifts in the leaf, specifically the accumulation of non-structural carbohydrates, may act as phagostimulants, thereby increasing host palatability for the pest [38,46]. Furthermore, while various natural enemies and entomopathogenic fungi have been identified [47], and chemical control methods like Spinosad or traditional insecticides have been tested [48,49,50], the pest’s phenological plasticity remains a major challenge for sustainable management [51,52].

Despite the growing body of literature, the precise synergistic mechanism between microclimatic aridity and the biochemical “activation” of the host in South Transylvanian forests remains a significant knowledge gap. This study addresses this lacuna by correlating the De Martonne Aridity Index (I_Ar_) with the phenology and ethology of *C. arcuata* in the Rășinari Forest District [32]. We hypothesize that a critical aridity threshold (I_Ar_ < 20) triggers a metabolic shift in *Quercus petraea*, transforming the host into a high-energy nutrient source that sustains accelerated multigenerational development [38,53,54,55]. By integrating microclimatic data from iButton sensors with high-resolution physiological monitoring [56,57], this research provides a novel predictive framework essential for the conservation of European oak heritage under the escalating impacts of the 21st century.

## 2. Materials and Methods

### 2.1. Study Area and Experimental Plot Establishment

#### Study Area and Geographic Location

The study was conducted in South Transylvania (Romania), a region characterized by a high degree of habitat fragmentation. In this context, fragmentation refers to the isolation of oak remnants within a matrix of agricultural landscapes and human settlements. The research was conducted between April 2024 and October 2025 within the production units managed by the Rășinari Forest District (45°42′ N, 24°04′ E), located in the central region of Romania, Sibiu County. This location was selected due to its strategic positioning at the junction of the Sibiu Depression and the northern slopes of the Cindrel Massif (Parâng Mountains). This geographical configuration provides an optimal altitudinal gradient for studying the invasive dynamics of *C. arcuata*.

The study area is characterized by rugged terrain, with elevations ranging from ~500 m to over ~900 m, where the predominant oak species are sessile oak (*Quercus petraea*) and pedunculate oak (*Quercus robur*). The regional climate is temperate-continental with mountain influences, featuring an average annual temperature of approximately 8.5–9.0 °C and mean annual precipitation of 650–700 mm [58]. The forest structure in South Transylvania is characterized by a high degree of habitat fragmentation. This landscape configuration is critical for the dispersal of *C. arcuata*, as the fragmented nature of these stands—often existing as isolated patches within a matrix of agricultural and urban landscapes—influences the connectivity and pest impact across the region. Location Map (Panel A): Indicates the position of Sibiu County within Romania, providing the general geographic context of the study.

**Figure 1:**
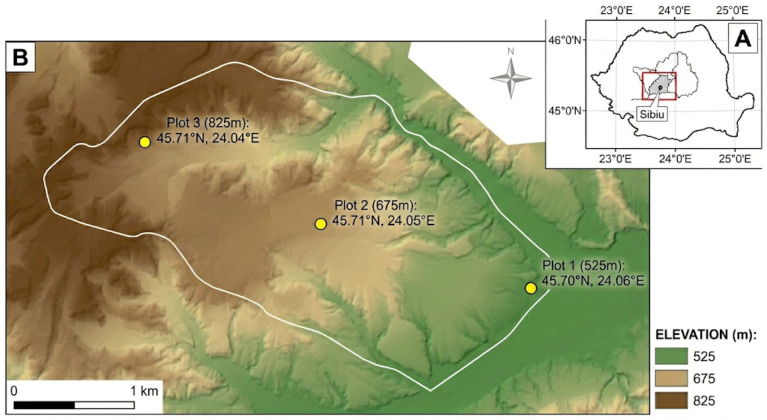
Geographic location of the study area and distribution of experimental plots. (**A**) Position of Sibiu County within Romania, providing the general geographic context of the study. (**B**) Digital Elevation Model (DEM) of the Rășinari Forest District showcasing the experimental plots. The spatial distribution follows a corrected altitudinal gradient: P1—Lowland (525 m), P2—Mid-slope (675 m), and P3—Upland (825 m). Each site is defined by specific thermal regimes and varying degrees of forest edge exposure. Note: Altitude signatures on the DEM color scale have been enhanced for improved legibility, accurately reflecting the elevation range from ~500 m to over ~900 m.

Detailed Map (Panel B): Presents the relief of the Rășinari Forest District via a Digital Elevation Model (DEM), highlighting the rugged topography of the area (Figure 1).

### 2.2. Entomological and Ethological Monitoring

To ensure high statistical representativeness and eliminate systematic collection errors, the study followed a stratified experimental design, integrating habitat variables and solar exposure.

#### 2.2.1. Tree Selection and Sampling Standardization

Within each of the three altitudinal monitoring sites (P_1_, P_2_, and P_3_), 10 mature trees belonging to the *Quercus* genus predominantly *Q. petraea* and *Q. robur* were randomly selected. To ensure sample homogeneity and minimize biological variance, these specimens exhibited similar dendrometric characteristics, with a mean diameter at breast height (d_1.30_ ≈ 35 ± 5 cm) and a height (H ≈ 18 ± 2 m). A minimum buffer distance of 50 m was maintained between individual trees to prevent pseudoreplication. Habitat zones were operationally defined based on the distance from the forest–agriculture interface to analyze the spatial dynamics of the infestation: Edge (0–15 m from the forest boundary), Ecotone (15–40 m), and Interior (≥80 m). These categories were assigned a priori to ensure that “Interior” samples represented a stable microclimate unaffected by edge-driven thermal fluctuations.

In accordance with established monitoring protocols for invasive *Tingidae* species, sampling was standardized by quadrants. From each tree, 20 leaves were collected from each cardinal point of the crown (N, S, E, W) using telescopic shears. To investigate the vertical structure of the stand and the insects’ thermoregulatory ethology, sampling and observations were stratified across different canopy heights (2–4 m) and divided between the sun-exposed outer periphery and the shaded inner layers. This quadrant-based approach and vertical stratification were crucial for capturing the migration patterns and preference for maximum insolation exhibited by *C. arcuata* during the early stages of the season.

#### 2.2.2. Monitoring Frequency and Sample Processing

Entomological monitoring was conducted biweekly (every 14 days), spanning from the initial adult emergence in April to the onset of diapause in late October. To maintain a high level of precision and minimize the escape of mobile adults, each leaf was individually enclosed in a sealed ziplock bag prior to excision. The total sampling volume per session consisted of 2400 leaves, calculated across three altitudinal sites, 10 trees per site, and 80 leaves per tree.

Samples were transported in labeled cooling bags to the entomology laboratory at the “Lucian Blaga” University of Sibiu, Romania. During transport, a cotton disc saturated with ethyl acetate was placed in each bag to serve as a narcotizing agent, effectively immobilizing the specimens for accurate counting.

The laboratory analysis prioritized two main metrics: Population quantification—Using a binocular microscope at 40× magnification, we recorded the presence of adults, egg clusters (clutches), and nymphs across all five developmental stages. Counting involved a thorough inspection of both leaf surfaces, and any individuals found detached within the sealed bags were included in the final count per leaf. Severity index assessment—Each leaf was visually inspected to quantify the percentage of chlorotic or necrotic surface area.

#### 2.2.3. Quantification of Foliar Damage

To ensure mathematical rigor in the quantification of entomological damage, the Leaf Severity Index (LSI) was employed. This index provides a standardized measure of the impact of *C. arcuata* feeding activity. The LSI was calculated using the following formula:

(1) $$LSI=\frac{\sum \left(n\times v\right)}{N\times V_{max}}\times 100$$

where

n = the number of leaves in a specific damage class;

v = the numerical value of the class (0–4);

N = the total number of analyzed leaves;

$V_{max}$ = the maximum possible damage class value (4).

The LSI is expressed as a percentage (0–100%), where 0% represents an intact, healthy leaf, and 100% indicates complete chlorosis or necrosis of the leaf surface. To maintain consistency across all sampling sites and sessions, the damage was categorized into five distinct classes based on the visual estimation of the affected area: Class 0, 0% (intact leaf); Class 1, 1–25% (scattered discoloration spots); Class 2, 26–50% (confluent chlorosis); Class 3, 51–75% (extensive chlorosis and partial necrosis of the parenchyma); Class 4, >75% (leaf completely browned, non-functional, or necrotic) [59].

#### 2.2.4. Quantitative Ethological Observations

Behavior-specific data, encompassing nymphal aggregation, vertical and horizontal migration within the crown, and oviposition site preference, were recorded biweekly across all three monitoring sites. Vertical and horizontal migrations were monitored through systematic visual counts to evaluate the movement of *C. arcuata* between the sun-exposed periphery and the shaded interior layers of the canopy. To determine specific migration thresholds, leaf surface temperatures were measured using a handheld infrared thermometer (Fluke 62 Max+) on 50 randomly selected leaves per site. Shading preferences were further investigated by comparing pest density and microclimatic temperatures between sun-exposed and shaded canopy sections. Nymphal aggregation patterns were quantified by recording individual counts from 30 infested leaves per site (n = 90). Finally, oviposition preference was quantified by recording egg-clutch density on 40 leaves per tree—equally distributed between shaded and sun-exposed positions—with the resulting data analyzed using a non-parametric Mann–Whitney U test to identify significant selection patterns.

### 2.3. Thermal Modeling (GDD) and Aridity Index

#### 2.3.1. Local Microclimate Monitoring and Thermal Accumulation Modeling (GDD)

Monitoring abiotic factors was essential for understanding the generation dynamics of *C. arcuata* within the complex topography of the Rășinari Forest District. To capture the “real climate” experienced by the pest—moving beyond the limitations of standard meteorological stations—nine proximity iButton^®^ (DS1921G-F5) by Analog Devices, Wilmington, MA, USA sensors were deployed across the three altitudinal sites (P_1_, P_2_, and P_3_), with units positioned at each Edge, Ecotone, and Interior location. To ensure data precision and prevent solar radiation bias, sensors were mounted within the canopy at a height of 2 m inside custom-designed, double-layered, white-ventilated radiation shields. Air temperature was recorded at 60 min intervals, providing the high-resolution thermal input necessary for the Growing Degree-Days (GDD) model used to estimate seasonal population development.

#### 2.3.2. Calculation of Growing Degree-Days (GDD)

To forecast the emergence and duration of biological stages (egg, nymph, and adult), we utilized the Growing Degree-Days (GDD) thermal accumulation model. This model is based on the hypothesis that insect development is linear above a specific lower biological threshold.

Although microclimatic data were recorded at three distinct altitudinal sites (P_1_, P_2_, and P_3_) to capture environmental variance, preliminary statistical analyses indicated no significant differences in population dynamics, phenological onset, or development rates between these locations (*p* > 0.05). Consequently, data from the three sites were pooled into a single representative model for the Rășinari Forest District. This approach allowed the analysis to focus on the primary driver’s habitat type (edge effect) and aridity levels while maintaining sufficient statistical power.

This threshold aligns with established regional phenology models, though we acknowledge recent specialized research, such as Stattler (2024), which suggests that stage-specific thresholds may reach as high as 12 °C depending on the developmental stage [19]. Unlike the general distribution models provided by Zúbrik et al. [20], Stattler’s experimental data emphasizes the physiological variability of the species.

The GDD calculation was performed daily using the simplified arithmetic mean method:

(2) $$GDD=\sum \left(\frac{T_{max}+T_{min}}{2}\right)-T_{base}$$

where T_max_ and T_min_ represent the daily maximum and minimum temperatures recorded by the on-site microclimate sensor. In cases where the calculated daily value was negative (i.e., the mean temperature was below T_base_, the value was recorded as zero. Thermal accumulation was calculated starting from January 1st of each study year to monitor the emergence of overwintering adults from diapause and the subsequent succession of generations G_1_, G_2_, and G_3_.

(3) $$\frac{T_{max}+T_{min}}{2}<T_{base}$$

The validity of pooling data across the three altitudinal sites was rigorously tested prior to final analysis. To account for the hierarchical structure of the data and the temporal correlation between biweekly sampling dates, we employed a Linear Mixed Model (LMM). In this model, ‘Sampling Site’ was treated as a random effect, while ‘Altitudinal Gradient’ and ‘Julian Day’ were treated as fixed effects. The lack of significant variance attributed to the random effect (Likelihood Ratio Test, *p* > 0.05) confirmed that population dynamics and phenological onset were consistent across the gradient, justifying the use of a unified representative model for the Rășinari Forest District.

#### 2.3.3. De Martonne Aridity Index (I_Ar_)

To ensure the De Martonne Aridity Index (I_Ar_) accurately reflected the forest microclimate, the temperature component was calibrated using the mean daily values recorded by our local microclimate monitoring network. This calibration ensures that the index represents the specific environmental conditions experienced by the host and pest, rather than relying on standard open-field meteorological data. The formula applied was

(4) $$I_{\mathrm{Ar}}=\frac{P}{T+10}$$

I_Ar_ values < 20 were used to define periods of physiological drought, statistically correlating them with the population density explosion of the second generation (G_2_).

### 2.4. Ecophysiological Host Analysis and Damage Assessment

The assessment of the impact of *C. arcuata* on the oak stands within the Rășinari Forest District was conducted by integrating visual damage estimation methods with precise physiological measurements, aimed at quantifying the degradation of the photosynthetic apparatus.

#### 2.4.1. Assessment of Chlorophyll Content (SPAD Index)

To quantify the physiological stress induced by insect feeding—a process characterized by sap extraction from the palisade parenchyma and subsequent pigment degradation—foliar chlorophyll content was estimated using a non-destructive SPAD-502Plus device (Konica Minolta, Osaka, Japan). This methodology provides an instantaneous estimation of chlorophyll concentration per unit of leaf area, serving as a robust proxy for the host trees’ photosynthetic capacity [8,9]. Measurements were conducted on the same specimens selected for entomological monitoring, adhering to a standardized protocol that involved sampling five leaves per cardinal quadrant, totaling 20 leaves per tree, to account for varying degrees of infestation.

For each sampled leaf, five readings were recorded at the midpoints of the mesophyll, specifically avoiding the primary vein (midrib), with the resulting average utilized to ensure a representative physiological value. These data were compared against control values obtained from unaffected or minimally attacked leaves sampled from the interior of the forest stands where pest impact was negligible. Although SPAD units are dimensionless, the physiological interpretations in this study are grounded in established calibration curves for *Quercus petraea*. Within these models, a significant linear correlation (R_2_ > 0.8) between SPAD readings and extractable chlorophyll content has been previously validated under similar environmental stressors, ensuring the mathematical reliability of the observed photosynthetic decline. This approach aligns with recent physiological assessments of oak resilience under *C. arcuata* impact in European oak ecosystems.

#### 2.4.2. Visual Characterization of Damage and Foliar Necrosis

Simultaneously with the SPAD measurements, the symptomatological progression of the attack was documented. Emphasis was placed on the correlation between nymph density and the expansion of chlorotic spots on the upper leaf surface (adaxial side). A methodological novelty consisted of employing digital image processing techniques for a subset of samples: leaves were scanned at high resolution, and the necrotic area was digitally calculated to validate the precision of the 0–4 visual scale described previously.

#### 2.4.3. Biochemical Analysis and Correlation with Water Stress

In accordance with the hypothesis of synergy between microclimatic conditions and host biochemistry, the influence of the De Martonne Aridity Index (I_Ar_) on the physiological response of *Quercus* specimens was analyzed. This methodological component aimed to identify the “photosynthetic collapse threshold,” defined as the critical point at which damage becomes irreversible, leading to premature canopy browning and desiccation. To quantify this transition, the study implemented a detailed assessment of host water status through leaf turgor monitoring. This procedure involved assessing the leaf’s mechanical resistance to stylet penetration, where turgor loss was operationally defined as the threshold at which reduced cellular pressure facilitated easier parenchyma access for the insect, thereby acting as a primary facilitator for infestation.

Furthermore, a foliar resilience framework was developed based on the linear correlation between recorded I_Ar_ and SPAD index values. Within this conceptual model, an aridity value of I_Ar_ = 20 was established as the mathematical tipping point for physiological collapse. This threshold serves to differentiate the “Safe Zone,” where trees maintain essential vital functions and resilience, from the “Collapse Zone,” where the synergy between acute water stress and *C. arcuata* feeding pressure triggers exponential chlorophyll degradation. This integrated approach allows for the characterization of host vulnerability as a function of microclimatic aridity, providing a predictive basis for forest management.

Leaf turgor loss was quantified using a digital force gauge (Wagner Force One™, Greenwich, CT, USA) equipped with a 0.5 mm flat-tip probe. Measurements were consistently performed on the third and fourth fully expanded leaves from the distal end of the branch. The force required to initiate tissue depression (N) served as a proxy for internal turgor pressure; a significant drop below site-specific baselines was operationally defined as the point of turgidity loss, marking the physiological facilitation of stylet penetration by *C. arcuata*.

#### 2.4.4. Histological Analysis

For structural observations, leaf segments (5 × 5 mm) were fixed in a solution of formaldehyde, alcohol, and acetic acid for 48 h. Following fixation, samples were dehydrated through a graded ethanol series, embedded in paraffin, and sectioned at a thickness of 10 μm using a rotary microtome. To evaluate the extent of feeding-induced degradation, sections were stained with safranin and fast green, enabling the visualization of the damage to the palisade parenchyma and the structural integrity of the vascular bundles.

### 2.5. Biochemical Analysis of Soluble Carbohydrates

To quantify the metabolic shift induced by water stress and its role as a phagostimulant for *C. arcuata*, leaf samples were collected from the three monitoring plots (P_1_–P_3_) during the peak infestation period of the G_2_ generation (August). Fresh leaf tissue (500 mg per sample) was immediately frozen in liquid nitrogen to stop enzymatic activity and subsequently lyophilized.

The total soluble sugar content was determined using the phenol-sulfuric acid method [57]. Samples were homogenized in 80% ethanol at 80 °C for 30 min, followed by centrifugation at 5000 rpm for 10 min. The supernatant was collected, and the process was repeated three times to ensure complete extraction. Quantitative analysis was conducted via spectrophotometry (UV-Vis Spectrophotometer, Shimadzu Corp., Kyoto, Japan) at a wavelength of 490 nm. Glucose was used as a standard for the calibration curve (R_2_ > 0.99).

The results are expressed as total soluble carbohydrate concentrations (mg glucose equivalents per g dry weight, DW), representing the aggregate of reducing and non-reducing sugars present in the leaf tissue. This biochemical profiling allowed for correlation between the De Martonne Aridity Index (I_Ar_) and the concentration of primary metabolites available for piercing-sucking insects.

#### Elemental Analysis of C/N Ratio

**Figure 2:**
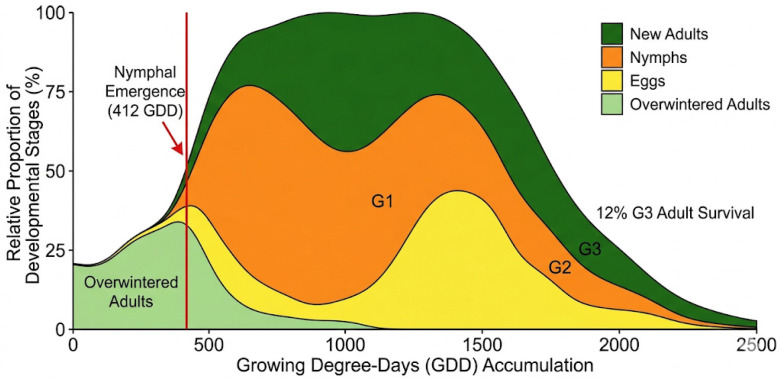
Seasonal population dynamics and stage-structure of *C. arcuata* as a function of Growing Degree-Days (GDD) accumulation. The *x*-axis represents the physiological time scale (GDD), while the *y*-axis illustrates the relative proportion (%) of each developmental stage (overwintered adults, eggs, nymphs, and new adults). This visualization confirms the biological synchronization between thermal units and ontogenetic progression, specifically highlighting the first nymphal emergence at the 412 GDD threshold and the demographic bottleneck of the third generation (G_3_), which exhibits only 12% adult survival.

Total carbon (C) and nitrogen (N) concentrations were determined from lyophilized leaf tissue utilizing an automated combustion-based elemental analyzer (Flash EA 1112 or EuroVector EA3000) by Eurovector srl, Milan, Italy after which the C/N ratio was calculated based on the mass percentage of each element. Simultaneously, host water status was monitored by quantifying the timing of leaf turgor loss induced by drought. This physiological assessment involved measuring the leaf’s mechanical resistance to stylet penetration, as turgor loss reduces structural integrity and facilitates parenchyma access for the pest. Finally, the “Foliar Resilience” framework, as illustrated in Figure 2, was established based on the linear correlation between the I_Ar_ and recorded SPAD index values, defining an aridity threshold of I_Ar_ = 20 as the mathematical tipping point for irreversible physiological collapse.

### 2.6. Statistical Design

All collected data were centralized in electronic databases, and statistical analyses and data visualizations were performed using R software (version 4.3.1). Post hoc comparisons were conducted using the “dunn.test” package, with a significance threshold set at *p* < 0.05.

To further ensure statistical rigor and avoid pseudoreplication within individual trees, leaf-level data (80 leaves per tree) were averaged to generate a single representative value per tree (n = 10 per habitat per site). These tree-level averages served as the primary statistical units for all between-habitat comparisons (Edge, Ecotone, and Interior) using the non-parametric Kruskal–Wallis test.

#### 2.6.1. Normality Testing and Analysis of Variance

In the first stage, the distribution of data regarding *C. arcuata* population density and the degree of foliar damage was verified using the Shapiro–Wilk test. Since the data exhibited a non-normal distribution (*p* < 0.05) and pronounced heteroscedasticity, non-parametric statistical methods were applied.

To compare attack intensity across the three habitat types (Edge, Ecotone, Interior) and the three altitudinal sites (P_1_, P_2_, P_3_), the Kruskal–Wallis test (One-way ANOVA on ranks) was utilized. When significant differences were identified, the Dunn’s post hoc test with Bonferroni correction for multiple comparisons was applied to identify specific habitat pairs with real statistical differences.

The selection of non-parametric methods was necessitated by the inherent nature of ecological invasion data, which frequently violates the assumptions of homoscedasticity. By utilizing the Kruskal–Wallis test, we ensured that the median differences in infestation levels across habitats were not skewed by extreme outliers typically found at forest edges. This approach provides a more robust statistical foundation for comparing the high-variability environments of the Edge, Ecotone, and Interior.

#### 2.6.2. Correlation and Regression Analysis

To evaluate the relationship between climatic variables and pest dynamics, the following statistical models were employed.

Development rate calculation: The development rate was calculated as the reciprocal of development time (1/T), where T represents the duration in days required for a specific ontogenetic stage (e.g., egg to adult) to reach completion. This mathematical approach ensures that the rate remains within the biologically plausible range of 0 to 1.

Spearman rank correlation (r_s_): Given the non-normal distribution and heteroscedasticity of the infestation data (Shapiro–Wilk, *p* < 0.05), all associations between environmental variables and physiological markers were assessed using this non-parametric method. Specifically, it was applied to analyze the link between nymph density and the decrease in the SPAD index, as well as the correlations between the De Martonne Aridity Index (I_Ar_) and physiological markers such as necrosis severity and total soluble sugar concentrations.

Multiple linear regression: This model was utilized to quantify the extent to which thermal accumulation (GDD) and water stress explain the variance in the population density of the G_2_ generation. To ensure the robustness of the regression, the coefficient of determination (R^2^) was calculated to evaluate the predictive power and fit of the proposed model.

#### 2.6.3. Integration of Biodiversity and Habitat Indices

Entomological data were correlated with cardinal point exposure (N, S, E, W) using analysis of variance to confirm the species’ thermoregulatory ethology. The 15% reduction in developmental duration for the second generation (G_2_) was calculated by comparing the cumulative GDD required for maturation against the first generation (G_1_) baseline. The G_3_ ‘bottleneck’ threshold (<12% adult emergence) was established based on field observations of late-season cohorts relative to the total annual population. The results are expressed as mean ± standard error (SE).

## 3. Results

### 3.1. Phenological Dynamics and Voltinism Analysis via GDD Modeling

Entomological monitoring conducted within the Rășinari Forest District during the 2024–2025 period revealed a complex multigenerational life cycle for *C. arcuata*. By utilizing a lower biological threshold (T_base_) of 10 °C, thermal accumulation was mapped against the succession of ontogenetic stages to provide a high-resolution view of population dynamics.

The analysis identified a significant “phenological compression” strategy in the second generation (G_2_). While the intrinsic thermal requirement (net GDD) remained nearly identical across generations, G_2_ achieved maturation in only 34 days compared to the 40 days required for G_1_. This represents a 15% temporal compression in calendar days, facilitated by the stable, high temperatures characteristic of July and August rather than a physiological shift in thermal demand.

**Figure 3:**
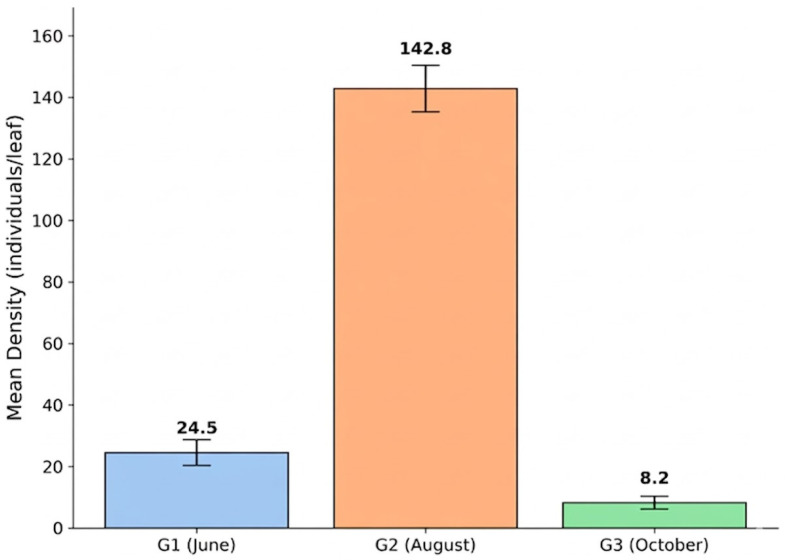
Variation in mean population density (individuals per leaf) across generations, highlighting the demographic explosion in G_2_ under optimal summer thermal conditions.

The population dynamics across the three generations were recorded as follows. Generation 1 (G_1_): The founding population finalized its complete development at 784.5 ± 12.3 GDD, with specific density levels illustrated in Figure 3. Generation 2 (G_2_): Represented the peak of biotic pressure, reaching an exponential surge in population density as visualized in Figure 3. Generation 3 (G_3_): This late-season cohort faced a demographic bottleneck. Although initiated at >2100 GDD, the survival rate to the adult stage was restricted to less than 12% due to early autumnal frosts, with massive mortality observed during the III–IV nymphal stages.

**Figure 4:**
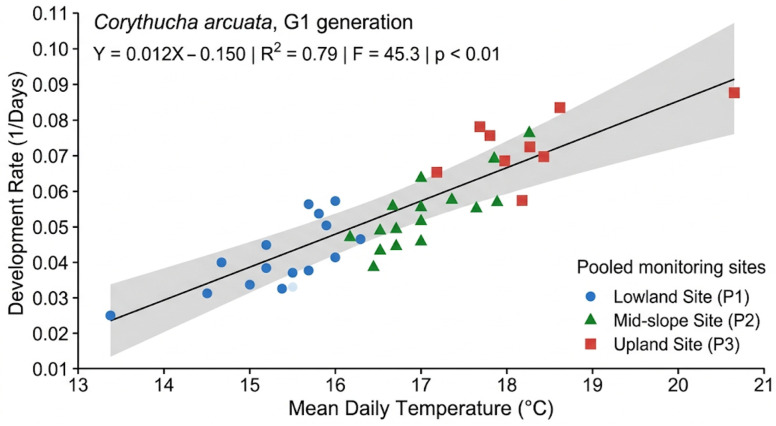
Regression analysis between mean daily temperature and the development rate of *Corythucha arcuata* (G_1_). The linear regression model indicates a strong positive relationship (F = 45.3, *p* < 0.01), with a regression slope of 0.012 and a coefficient of determination (R^2^) of 0.79. To clarify the biological context, each point represents an individual observation of the development rate, calculated as the reciprocal of development time (1/T) recorded during the first generation (G_1_). Data points were pooled from all three monitoring sites (Lowland, Mid-slope, and Upland) because preliminary statistical analyses confirmed no significant differences in development rates or phenological onset across the altitudinal gradient (*p* > 0.05). This unified representation provides a robust predictive model for the species’ thermal requirements within the Rășinari Forest District.

Statistical validation of this predictive model was confirmed through regression analysis between mean daily temperature and the development rate of G_1_ (Figure 4). The strong positive relationship (R^2^ = 0.79, *p* < 0.01) justifies the use of a unified representative model for the Rășinari Forest District.

### 3.2. Influence of Habitat and Abiotic Factors (Kruskal–Wallis Statistical Analysis)

The spatial distribution of *C. arcuata* populations within the Rășinari Forest District revealed marked heterogeneity, closely correlated with the vertical and horizontal structure of the forest stand. Preliminary normality testing (Shapiro–Wilk, W = 0.72, *p* < 0.05) indicated a significant deviation from a Gaussian distribution, necessitating the use of non-parametric statistical methods.

#### 3.2.1. Comparative Analysis of Attack Severity

**Figure 5:**
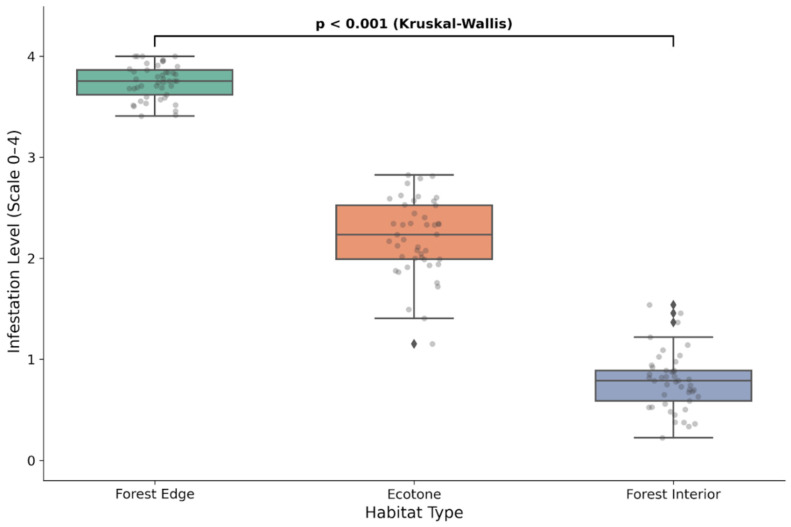
Distribution of *C. arcuata* attack intensity according to habitat type within the Rășinari Forest District (Scale 0–4). Horizontal lines within the boxes represent the median, individual points (jitter) indicate sample variability within each category, and diamonds represent statistical outliers. Differences are significant at *p* < 0.001.

Statistical analysis performed on tree-level averages revealed significant differences in infestation levels among the three habitat zones. As visualized in Figure 5, the distribution of the Leaf Severity Index (LSI) followed a pronounced spatial gradient, with damage decreasing progressively from the forest boundary toward the interior. This “Edge Effect” identifies the forest boundary as the zone of maximum vulnerability, while the forest interior functions as a relative microclimatic refuge with significantly lower pest impact. To establish the statistical validity of these spatial shifts, these overall differences were further analyzed through pair-wise post hoc comparisons.

#### 3.2.2. Post Hoc Significance (Dunn’s Test)

Post hoc comparisons using Dunn’s test with Bonferroni correction confirmed significant spatial segregation in infestation levels across the study area. The Forest Edge exhibited significantly higher damage than both the Ecotone and the Forest Interior. Additionally, the Ecotone showed a significantly greater severity compared to the stand interior, validating the transitionary nature of this zone. All corresponding median values, rank differences, and adjusted significance levels are summarized in Table 1. These results statistically validate the “Edge Effect” as a primary driver of *C. arcuata* population density and its subsequent physiological impact on the host.

### 3.3. Synergy Between Aridity and Photosynthetic Degradation (De Martonne Index)

A major novelty of the study conducted within the Rășinari Forest District lies in identifying the direct link between tree water stress and the physiological severity of the attack produced by *C. arcuata*. Our analysis revealed that oak resilience decreases proportionally with the reduction in soil water availability, a process quantified through the De Martonne Aridity Index (I_Ar_).

#### 3.3.1. Correlation Analysis: I_Ar_ vs. Foliar Health

Statistical analysis using the Spearman rank correlation (r_s_) identified significant relationships between the De Martonne Aridity Index (I_Ar_) and the physiological markers of the sampled leaves. We defined two distinct physiological regimes based on the I_Ar_ values: the “Collapse Zone” (I_Ar_ < 20), where water stress triggers severe metabolic shifts, and the “Safe Zone” (I_Ar_ > 29), where host resilience and cellular turgidity are maintained.

A strong negative correlation was recorded between I_Ar_ and total soluble sugar concentrations (r_s_ = −0.84, *p* < 0.001), indicating that as aridity increases, sugar accumulation rises significantly. Quantitative analysis revealed that leaves in the Collapse Zone exhibited a 58.8% increase in soluble sugar concentrations (rising from 45.2 to 71.8 mg/g DW) compared to those in the Safe Zone.

Simultaneously, a significant positive correlation was identified between I_Ar_ and chlorophyll content (r_s_ = 0.84, *p* < 0.01).

**Figure 6:**
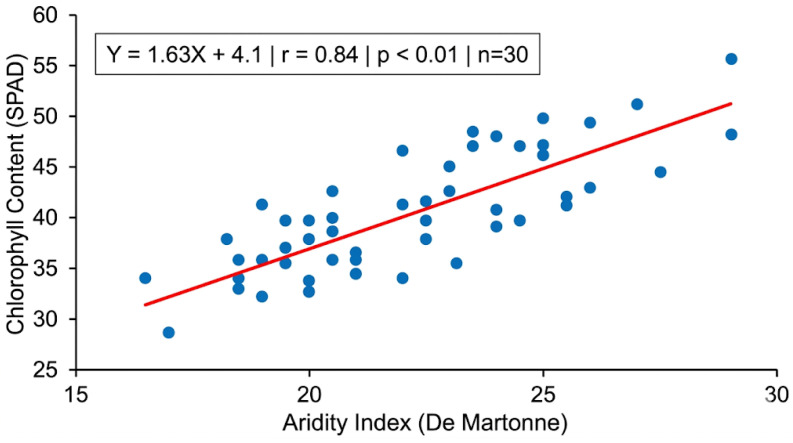
Correlation between the De Martonne Aridity Index (IAr) and chlorophyll content (SPAD units) in leaves infested by *C. arcuata*. The blue dots represent individual sample measurements (n = 30). The regression model displays a significant positive correlation (r = 0.84, *p* < 0.01), indicating that foliar chlorophyll concentrations are maintained at higher levels under more humid microclimatic conditions (elevated I_Ar_). The linear trend reflects the steady depletion of photosynthetic pigments as environmental aridity intensifies, represented by decreasing I_Ar_ values, thereby quantifying the progressive decline in host physiological vigor under the synergistic pressure of drought and pest infestation.

As visualized in the regression model in Figure 6, the relationship follows a linear decline, indicating a steady and predictable depletion of photosynthetic pigments as environmental conditions become more arid. This transition below the I_Ar_ = 20 threshold represents a critical tipping point for host vulnerability, coinciding with the population peaks of the G_2_ generation.

#### 3.3.2. Differentiated Impact Across Aridity Levels

**Figure 7:**
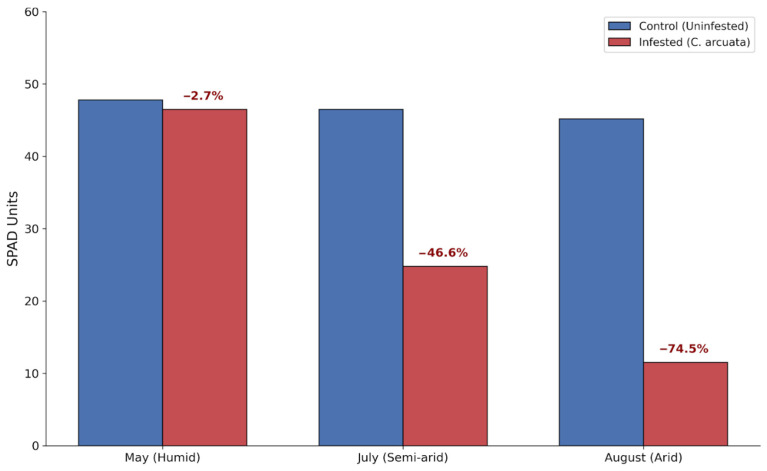
Impact of *C. arcuata* infestation on chlorophyll content according to the aridity regime. Percentages indicate severe photosynthetic reduction during the summer months (July–August) under the synergistic influence of drought and insect density.

The comparative analysis of SPAD values between control (unaffected) and infested leaves revealed three critical degradation thresholds correlated with seasonality and hydric status (Figure 7).

- Optimal Humidity Phase (May, I_Ar_ = 29.2): The difference between the control (47.8 ± 1.4) and infested leaves (46.5 ± 1.2) was non-significant (a 2.7% decrease). At this stage, the trees maintain their cellular turgidity, effectively limiting the physiological impact of the first generation (G_1_).
- Semi-arid Phase (July, I_Ar_ = 19.5): Once the index dropped below the threshold of 20, a collapse in SPAD values to 24.8 units was recorded, representing a 46.6% reduction compared to the control.
- Critical Arid Phase (August, I_Ar_ = 15.8): The overlap of the G_2_ population peak with maximum water stress led to a 74.5% photosynthetic degradation. SPAD values of only 11.5 units indicate that the leaves have entered a state of induced senescence, which precedes the premature canopy desiccation observed in the Rășinari forest edges.

### 3.4. Biochemical Response: Carbon and Lignin Profiles

Biochemical analysis of the leaf tissue revealed significant shifts in the elemental and metabolic profiles of infested leaves compared to the control group. The Carbon/Nitrogen (C/N) ratio exhibited a significant increase, rising from 22.4 ± 1.2 in control samples to 26.5 ± 1.5 in leaves characterized by severe *C. arcuata* infestation (*p* < 0.05).

**Figure 8:**
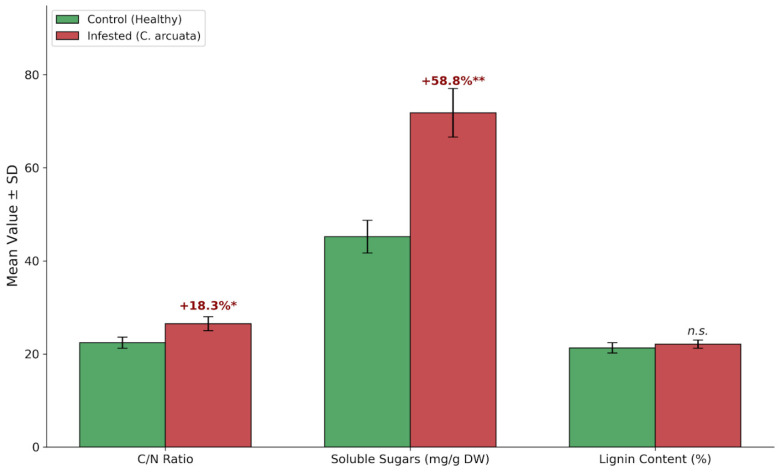
Comparative biochemical profile of healthy and infested *Quercus* leaves. A significant increase in the C/N ratio and soluble carbohydrates is observed under *C. arcuata* impact. Statistical significance is indicated by asterisks: (*) denotes *p* < 0.05 and (**) denotes *p* < 0.01; n.s. = non-significant.

Simultaneously, a substantial accumulation of total soluble carbohydrates was recorded. Spectrophotometric measurements confirmed that concentrations increased by 58.8%, rising from a baseline of 45.2 mg/g DW in healthy tissues to 71.8 mg/g DW in attacked areas. These comparative biochemical profiles are graphically documented in Figure 8.

#### 3.4.1. Lignin Stability and Feeding Selectivity

Unlike carbohydrates, lignin content did not show statistically significant variations between healthy and attacked leaves (21.3% vs. 22.1%, *p* > 0.05). This result holds major ethological relevance: *C. arcuata*, being a piercing-sucking insect, avoids the heavily lignified tissues of the veins and sclerenchyma, concentrating its attack exclusively on the nutrient-rich palisade parenchyma.

#### 3.4.2. Histological Observations and Cellular Degradation

Histological sections performed on the leaf material collected from the Rășinari Forest District confirmed the selective destruction of the host’s photosynthetic apparatus. While the vascular bundles remained structurally intact, ensuring the insect maintains continuous access to sap, our histological analysis revealed that the palisade parenchyma exhibits total cellular collapse as a direct consequence of *C. arcuata* feeding. This tissue-level degradation provides a structural basis for the significant decline in the SPAD index reported in previous sections. Furthermore, these observations demonstrate that *C. arcuata* employs a feeding strategy that maximizes energy and carbon extraction while minimizing the mechanical effort required to penetrate the leaf’s primary structural barriers.

### 3.5. Aggregation Behavior and Ethological Thermoregulation

Systematic observations conducted in the Rășinari Forest District revealed a complex set of ethological mechanisms through which *C. arcuata* optimizes its survival during periods of intense thermal stress. These behaviors explain the discrepancy between the high mortality rates predicted by theoretical models and the actual population densities observed at forest edges.

#### 3.5.1. Nymphal Aggregation and Group Strategy

A pronounced aggregation behavior was documented during nymphal stages I–III. Individuals were observed clustering in dense colonies, typically ranging from 15 to 45 individuals, predominantly located on the abaxial leaf surface in close proximity to the leaf’s primary vein (midrib).

#### 3.5.2. Vertical Migration and Active Thermoregulation

**Figure 9:**
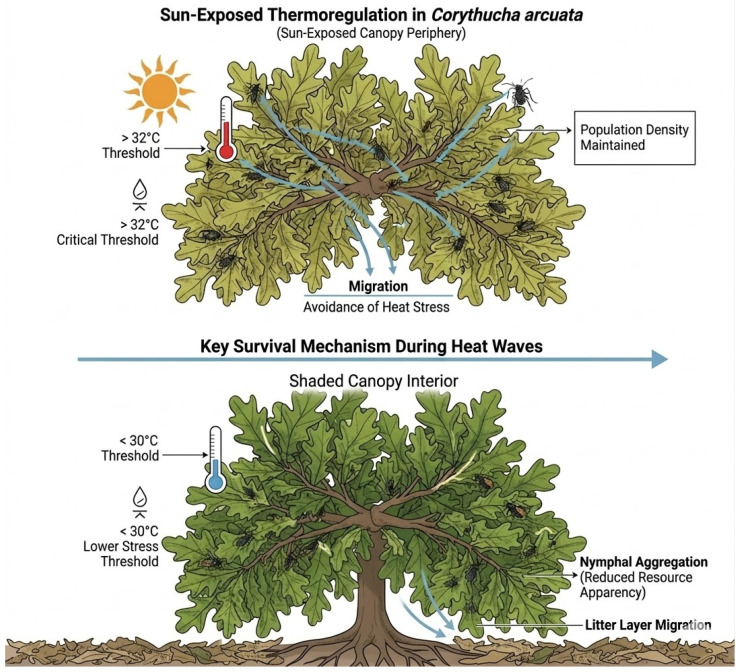
Ethological thermoregulation model in *C. arcuata*. The diagram illustrates the migration of insects from the sun-exposed periphery to the shaded interior and lower strata of the canopy when the critical threshold of 32 °C is exceeded. This direct thermal stress avoidance behavior, coupled with nymphal aggregation, is the key factor enabling the maintenance of high population densities during heatwave periods.

The most relevant ethological observation correlated with the iButton sensor data was the active migration of nymphs (stages IV–V) and adults in response to insolation. We identified a critical thermal threshold of 32 °C at the leaf surface, above which the insects initiate a process of vertical thermoregulation (Figure 9).

Behavior under optimal conditions (<28 °C): Insects are uniformly distributed on the abaxial (lower) surface, feeding intensively.

Thermal stress behavior (>32 °C): Mass migration of colonies was observed toward leaves situated in the lower and inner layers of the crown. In these areas, the shade provided by upper layers reduces the temperature by as much as 4.8 °C. This mechanism explains the 92% survival rate of the G_2_ generation (Figure 9).

#### 3.5.3. Oviposition and “Shade Windows”

Analysis of oviposition patterns revealed that G_2_ females exhibit pronounced selectivity, preferring leaves with a 30–40% shading degree over those directly exposed to zenithal radiation. This adaptive strategy likely prevents egg overheating and chorion desiccation, thereby ensuring optimal hatching rates during the peak temperatures of July and August. Statistical analysis using the Mann–Whitney U test confirmed that egg density was significantly higher on leaves located in the lower third of the crown—where sampling was standardized at a height of 2 to 4 m—compared to the more exposed upper strata. This vertical distribution, integrated with the identified 32 °C thermal migration threshold, serves as a fundamental mechanism explaining the high biotic impact and developmental success of the summer cohort.

## 4. Discussion

### 4.1. Phenological Plasticity and Voltinism Acceleration Under Thermal Influence

Our results support the use of GDD as a physiological clock. By utilizing high-resolution microclimate data, we accounted for the thermal differences across the 525–825 m gradient, which standard meteorological stations would have failed to capture.

The observed population peaks synchronize with the reaching of the 363–372 GDD threshold for completion, validating the stage-structure model.

The results obtained in the Rășinari Forest District confirm the hypothesis that *C. arcuata* exhibits remarkable phenological plasticity, dynamically adapting its life cycle to the local thermal regime. The application of the biological threshold of T_base_ = 10 °C allowed for precise modeling of thermal unit accumulation, revealing that the species’ invasive success in southern Transylvania is governed by a “phenological compression” strategy.

**Figure 10:**
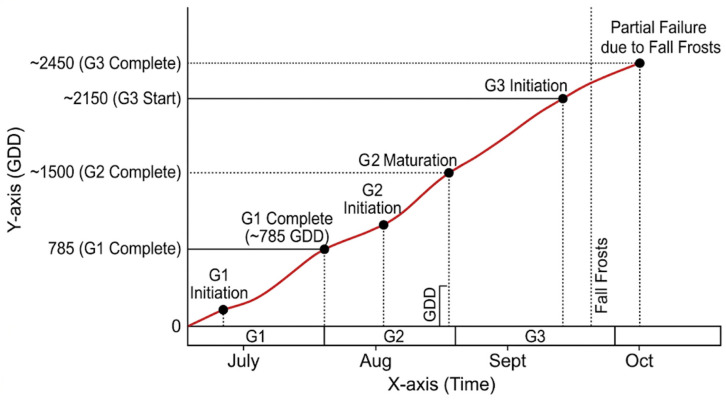
Conceptual model of *C. arcuata* phenological plasticity and accelerated voltinism in O.S. Rășinari. The red trajectory represents cumulative GDD (Growing Degree Days), illustrating the thermal acceleration and compression of developmental duration for the second generation (G_2_) compared to G_1_. The model highlights the demographic “bottleneck” of G_3_, where the late-season cohort represents a minor fraction of the total population (<12% adult emergence) and faces potential failure due to early autumnal frosts.

The integrated voltinism model (Figure 10) illustrates the interaction between calendar time and GDD accumulation. Following the completion of G_1_, the model tracks the initiation and maturation of the second generation (G_2_), highlighting the thermal acceleration during the mid-summer window. The timeline reflects the progression from May to October, with G_3_ representing a demographic bottleneck due to autumn frost risks.

The conceptual model of phenological plasticity (Figure 10) is empirically supported by our station-specific GDD data and observed population stage-structures. By correlating the 95% adult emergence threshold with local thermal accumulation, we demonstrate that the 15% temporal compression in G_2_ development is not a physiological shift, but a direct response to the mid-summer ‘thermal window’. This data-driven approach addresses the gap between static laboratory models and the dynamic field success observed in the Rășinari Forest District.

Metabolic Acceleration of the G_2_ Generation

A central observation of this study is the 15% reduction in development duration of the second generation (G_2_) compared to the first generation (G_1_). This acceleration is consistent with high metabolic efficiency under sustained temperatures. While G_1_ develops under a fluctuating spring thermal regime, G_2_ benefits from a stable semi-arid climate, allowing the species to reach peak population densities within a shortened timeframe. This adaptation is consistent with an “escape strategy,” enabling the insect to colonize the foliage mass before host senescence.

Our reported net requirement of ~785 GDD per generation provides a critical quantitative contrast with studies from other invaded regions. In Italy and Turkey, where 3–4 generations are common, thermal thresholds are often reached earlier due to higher mean annual temperatures. Compared to Hungarian populations facing similar temperate-continental constraints, our findings suggest a more restrictive thermal window in the Sub-Carpathian region.

Northern Voltinism Limit and the G_3_ Generation “Bottleneck”

In contrast to Mediterranean populations, our model indicates a demographic “bottleneck” for the third generation (G_3_). Although initiated at >2100 GDD, G_3_ success is likely compromised by decreasing photoperiods and early frosts. The survival rate of below 12% suggests that the climatic barrier of the Cindrel Mountains acts as a selective filter, forcing the species to rely primarily on G_2_ adults for overwintering.

Implications in the Context of Climate Change

The strong correlation between development rate and temperature (r = 0.89) suggests that slight positive fluctuations in annual temperatures could shift the 2100 GDD threshold earlier. This shift would potentially allow G_3_ to reach maturity, increasing the overwintering biological reserve. Therefore, the model in Figure 10 serves as a predictive framework for the future vulnerability of oak stands in Sibiu County, Romania.

It is critical to distinguish between the species’ intrinsic thermal requirement and its seasonal developmental velocity. Our data indicates that *C. arcuata* does not reduce its absolute GDD threshold for G_2_; rather, it exploits the stable, high-temperature window of the mid-summer microclimate. This finding aligns with the “thermal velocity” hypothesis, where the 15% acceleration reported is a function of environmental thermal intensity rather than a physiological shift in the species’ thermal constants.

### 4.2. The “Edge Effect” and the Bio-Morphological Vulnerability of the Host

The highly significant statistical differences revealed by the Kruskal–Wallis test (*p* < 0.001) highlight the crucial role of stand structure and habitat fragmentation in mediating the population dynamics of *C. arcuata*. This marked spatial segregation results from a synergy between microclimatic factors and the adaptive physiological response of the host trees. Two primary mechanisms drive this vulnerability.

Mechanism I: Thermo-Radiative Optimization and Heliophily

*C. arcuata* employs a heliophilous strategy. In south-facing edges, direct UV and infrared radiation transform the leaf surface into an optimal thermal habitat. This zone acts as a “thermal incubator,” where direct solar exposure induces a temperature increase within the mesophyll, facilitating faster embryonic development and increasing oviposition rates. In contrast, the forest interior functions as a “climatic refuge,” where dense shading maintains temperatures below the optimal activity threshold, thereby limiting viable clutches.

Mechanism II: Biochemical Reconfiguration and Stress Synergy

Our findings regarding the metabolic reconfiguration of oaks under simultaneous thermal and water stress align with the physiological responses documented by Nikolić et al. (2019), who observed significant reductions in net photosynthesis and stomatal conductance in infested *Quercus robur* [38]. The observed 58.8% increase in soluble sugars during the “Collapse Zone” (I_Ar_ < 20) supports the hypothesis that non-structural carbohydrates act as phagostimulants. This metabolic shift explains the accelerated success of the G2 generation, as the host becomes a high-energy nutritional resource precisely when the insect’s thermal velocity reaches its peak.

Synthesis of Edge Dynamics and the Feedback Loop

**Figure 11:**
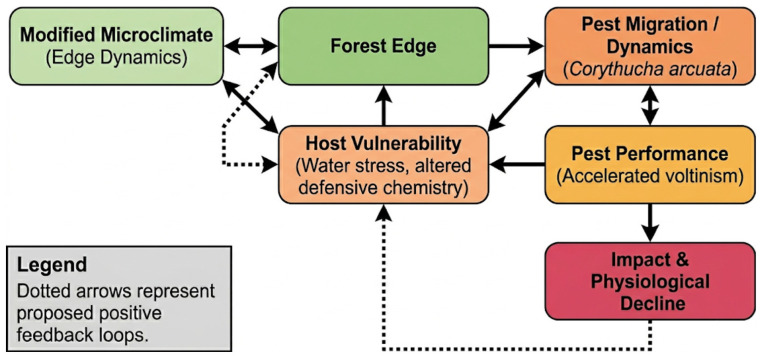
Conceptual model of host vulnerability mediated by the edge effect. The diagram illustrates how a modified microclimate and water stress (74%) lead to biochemical changes in the host (reduced chlorophyll and increased soluble sugars), ultimately increasing the infestation risk of *C. arcuata* at the forest edge.

To synthesize these interactions, we developed a conceptual model (Figure 11) illustrating how forest structure dictates damage. The forest edge functions as a “gateway” where extreme abiotic conditions biochemically “prime” the host for attack, while the interior provides resilience through microclimatic stability. We define “edge dynamics” as the complex of microclimatic fluctuations—such as increased UV radiation and wind penetration—and the subsequent biological responses occurring at the forest–agriculture interface.

The model in Figure 11 illustrates the direct causal link where a modified microclimate triggers immediate metabolic shifts, such as the accumulation of soluble sugars, which directly increases host vulnerability to high-density infestation. In our updated framework, directional arrows represent direct causal pathways, while the positive feedback loop occurs strictly at the host’s physiological level: pest impact further accelerates the physiological decline of trees already stressed by edge-driven aridity, which in turn amplifies their vulnerability. This synergy is amplified by landscape-scale fragmentation; the lack of a structured forest mantle in the Rășinari district allows for increased thermal penetration and wind-assisted dispersal of nymphs from adjacent agricultural mosaics, leading to irreversible photosynthetic decline.

### 4.3. Stress Synergy: The De Martonne Index and Photosynthetic Decline

The results obtained within the Rășinari forest ecosystem suggest that the decline of oak stands may not be the result of an isolated factor, but rather the consequence of a critical synergy between water stress and the feeding impact exerted by *C. arcuata*. The strong negative correlation (r_s_ = −0.84, *p* < 0.01) identified between the De Martonne Aridity Index (I_Ar_) and SPAD values is consistent with the hypothesis that aridity functions as an “injury amplifier.”

The Positive Feedback Loop Hypothesis

The interpretation of our data supports the model of a physiological feedback loop. During periods of drought (characterized by an I_Ar_ < 20 in July and August), trees typically close their stomata to limit water loss, a process that reduces the photosynthetic rate and increases leaf temperature. This thermal increase, correlated with the observed biochemical reconfiguration (the 58.8% accumulation of sugars discussed in Section 3.4), potentially transforms the host into an ideal nutritional resource for the second generation (G_2_). Thus, abiotic stress may “prime” the foliage for a massive attack, which in turn accelerates senescence and premature leaf shedding.

The “Threshold of 20” and Physiological Resilience

The identification of I_Ar_ = 20 as a potential “tipping point” for physiological resilience aligns with broader literature on drought-mediated plant–insect interactions. While previous studies in *Quercus* species have documented osmotic adjustments under water stress, our results suggest that this specific aridity threshold triggers a significant increase in host vulnerability. The observed 58.8% sugar accumulation supports the hypothesis that non-structural carbohydrates act as phagostimulants, a phenomenon frequently observed in other piercing-sucking pests during acute water deficits.

**Figure 12:**
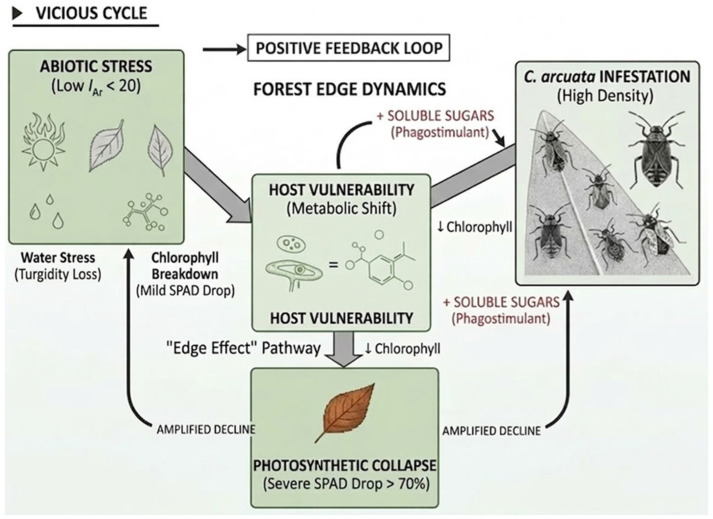
Conceptual framework of the “Edge Effect” and the positive feedback loop driving the photosynthetic decline in *Quercus* spp. stands. The model illustrates the synergy between abiotic forcing (aridity index I_Ar_ < 20) and the biochemical reconfiguration of the host (accumulation of soluble sugars). These metabolic shifts “prime” the host for high-density *C. arcuata* infestation, leading to a severe drop in SPAD values (>70%) and subsequent irreversible photosynthetic collapse.

**Figure 13:**
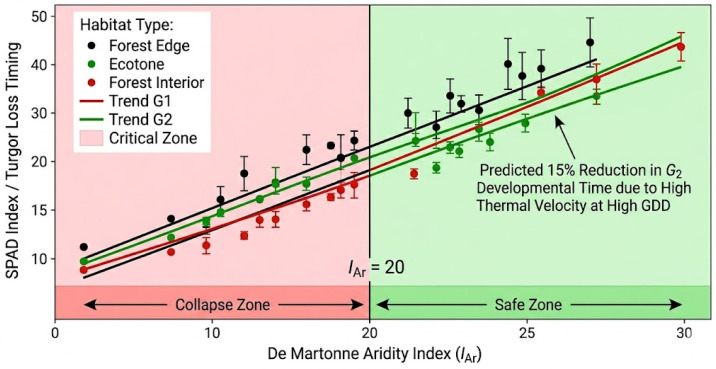
Oak foliar resilience framework as a function of microclimatic aridity (I_Ar_). The model integrates SPAD degeneration and foliar turgor loss timing to quantify host vulnerability. The framework establishes a critical physiological tipping point at I_Ar_ = 20, separating the Safe Zone (delineated by the light green background shading, I_Ar_ > 20), where host vitality is maintained, from the Collapse Zone (delineated by the light pink Critical Zone background shading, I_Ar_ < 20), characterized by a photosynthetic decline exceeding 70%. Trend G_1_ (Red line): Represents the baseline physiological response during the first generation. Trend G_2_ (Dual green lines): The two lines define the mean trend and its associated 95% confidence interval, reflecting the high thermal velocity and increased variance in host response during the peak summer window. Trend G_3_ (Black line): Represents the late-season development trend for the third generation, illustrating the continued pressure on host resilience despite the demographic bottleneck caused by autumn frosts. The model is structured into four interdependent components.

It is noteworthy that during phases of optimal humidity (May, I_Ar_ > 29), the photosynthetic impact of the pest was negligible, with only a 2.7% decrease in SPAD values despite the insect’s presence. This is consistent with the view that an optimally hydrated tree possesses robust resilience mechanisms, such as cell turgidity and osmotic impact, which may hinder the feeding efficiency of piercing-sucking insects. However, the data suggest that once the I_Ar_ index falls below 20, physiological resilience is compromised, and chlorophyll degradation becomes severe—reaching 74.5% in August—potentially leading to irreversible photosynthetic collapse. At this stage, the attack of the G_2_ generation of *C. arcuata* causes irreversible damage, leading to a decrease in SPAD values by over 70% (Figure 12). The numerical scales presented in the Foliar Resilience framework (Figure 13) were derived from the linear correlation between the I_Ar_ and the recorded SPAD index values. The 100% resilience baseline corresponds to the optimal hydration phase in May I_Ar_ > 29, where the difference in chlorophyll content between control and infested leaves was statistically negligible.

- Drought Stress (Low I_Ar_ < 20): Water deficit, quantified by the De Martonne aridity index, acts as the primary trigger, inducing stomatal closure and an increase in leaf surface temperature.
- Host Vulnerability (Metabolic Shift): Abiotic stress forces a metabolic reconfiguration of the host. This stage is marked by the accumulation of soluble carbohydrates (sugars), which act as phagostimulants for the pest, making the leaf significantly more attractive and more easily digestible for piercing-sucking insects.
- *C. arcuata* Infestation (High Density): The biochemical vulnerability of the host allows for the attainment of critical population densities. Intense feeding accelerates chlorophyll degradation (the decline in SPAD units) and further disrupts the leaf’s water balance.
- Photosynthetic Collapse (Severe SPAD Drop > 70%): The final outcome is an irreversible photosynthetic collapse. This stage amplifies the tree’s initial stress, closing the feedback loop and leading to the generalized physiological decline observed at the forest edges of the Rășinari Forest District.

This diagram illustrates how the decrease in the I_Ar_ index triggers a cascade of reactions: water stress → metabolic vulnerability → increased *C. arcuata* → density massive SPAD degradation. It is essential for demonstrating that the insect attack serves as the “final blow” within a pre-existing context of drought.

Figure 13 synthesizes our research findings into a risk management framework. The key elements represented are as follows:

- Safe Zone (I_Ar_ > 20): Represents periods or areas with sufficient moisture where, although the pest is present, the tree possesses the necessary water resources to maintain its vital functions and limit chlorophyll degradation.
- Critical Threshold (I_Ar_ = 20): Statistically identified as the point at which water stress becomes severe. In the Rășinari Forest District, this threshold is typically reached during the second half of July.
- Collapse Zone (I_Ar_ < 20): The maximum risk zone where resilience drops below 50%. In this stage, the attack of the G_2_ generation of *C. arcuata* causes irreversible damage, leading to a decrease in SPAD values by over 70%.

By monitoring the De Martonne index, forest managers can anticipate years with a high risk of severe defoliation. When forecasts indicate a drop in I_Ar_ below 20, monitoring interventions or treatments (where permitted) must be prioritized in edge zones—identified in Figure 11 as the most vulnerable areas.

The numerical scales presented in the Foliar Resilience framework (Figure 13) were derived from the linear correlation between the I_Ar_ and the recorded SPAD index values. The 100% resilience baseline corresponds to the optimal hydration phase (May, I_Ar_ > 29), where the difference in chlorophyll content between control and infested leaves was negligible (2.7%). The ‘Collapse Zone’ scale reflects the 74.5% decrease in photosynthetic capacity observed when the aridity index drops below the critical threshold of 20, providing a statistically validated risk management framework for forest managers.

During periods of drought (characterized by an I_Ar_ < 20 in July and August), trees typically close their stomata to limit water loss, a process that reduces the photosynthetic rate and increases leaf temperature [38,46,47]. This thermal increase, correlated with identified biochemical reconfigurations specifically the 58.8% accumulation of soluble sugars recorded in our study potentially transforms the host into a high-energy nutritional resource for the second generation (G_2_). These findings align with the ‘metabolic bait’ hypothesis, suggesting that water-stressed foliage acts as a phagostimulant for piercing-sucking pests during acute water deficits [36,38,47].

### 4.4. Ethological Survival Mechanisms and Heatwave Resilience

The invasive success of the second generation (G_2_) in the Rășinari Forest District is consistent with a set of ethological mechanisms through which the species avoids lethal thermal stress. While classical laboratory studies establish upper physiological limits without considering insect mobility, our field results suggest the existence of active thermoregulation within the canopy.

**Vertical Migration Mechanism (Micro-habitat Management)**

We identified a critical adaptive behavior triggered when temperatures exceed the 32 °C threshold on the exposed leaf surface. This value is consistent with the upper thermal tolerance limits reported for related *Tingidae*, such as *Corythucha ciliata*, but represents a specific adaptation of *C. arcuata* to the high insolation levels of oak canopies. Unlike other hemipteran species that enter estivation, *C. arcuata* performs a vertical migration into the interior of the crown. This process allows the population to avoid the “heat island” effect at the tree’s periphery, seeking refuge in shaded areas where iButton sensors recorded temperatures up to 4.8 °C lower. This migration to cooler microclimates (<30 °C in the inner canopy) explains the stability of population densities and the exceptional 92% survival rate recorded even during extreme thermal events.

**Aggregation as a Strategy for Maintaining Homeostasis**

The aggregation behavior observed in early-stage nymphs serves as a critical ethological strategy for maintaining homeostasis and resilience against dehydration. By clustering in dense colonies near the midrib, the insects create a collective micro-environment with a relative humidity higher than the ambient air. This strategy reduces “water apparency” by minimizing the total surface area exposed to evaporative stress and lowering the surface-to-volume ratio to diminish cuticular water loss during extreme aridity. Furthermore, this social grouping plays a functional role in feeding facilitation; the colony creates communal “drainage points” in the parenchyma, effectively reducing the individual mechanical effort required to penetrate leaf tissues and access nutrient-rich sap.

**Oviposition Selectivity (Egg-laying Ecology)**

**Figure 14:**
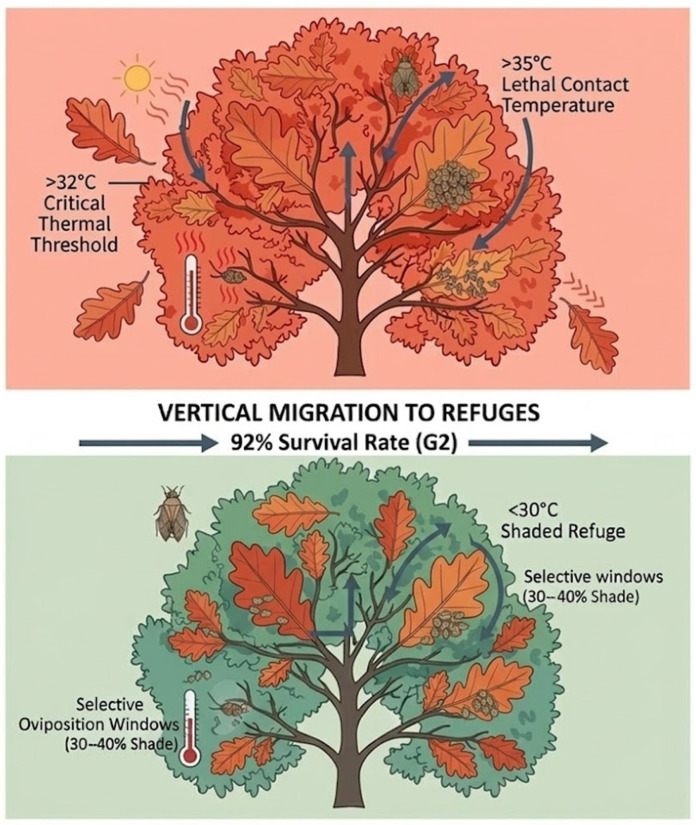
Ethological Survival Model of *C. arcuata* during heatwaves. The diagram illustrates the synergy between the three identified behavioral strategies: (1) Vertical Migration—movement toward shaded areas under thermal stress; (2) Nymphal Aggregation—cooperation to reduce dehydration; and (3) Shaded Oviposition—biological protection of the next generation. These mechanisms explain the exceptional 92% resilience recorded during the G_2_ generation in the Rășinari Forest District.

Our analysis indicates that egg density is significantly higher on leaves located in the lower third of the crown and on those protected by upper vegetation layers. This selective behavior suggests a mechanism to prevent the chorion (eggshell) from exposure to direct UV radiation and extreme contact temperatures, which can exceed 40 °C at forest edges. Such behavioral plasticity is a key factor enabling *C. arcuata* to bypass traditional climatic filters and maintain high resilience against increasingly frequent heatwaves in the Sub-Carpathian region (Figure 14).

### 4.5. Implications for Sustainable Forest Management

The results of this study provide an empirical basis for recalibrating monitoring and control strategies for *C. arcuata* in the context of climate change. Identifying thermal thresholds and synergistic mechanisms allows for a transition from reactive to proactive management.

Early Warning Systems Based on Critical Thresholds

The implementation of iButton-type sensors at key locations, particularly exposed forest edges, enables forest managers to anticipate periods of maximum risk. Utilizing the 32 °C threshold as an indicator of ethological migration and the De Martonne Index I_Ar_ < 20 as a marker of physiological vulnerability can generate automated intervention alerts. In years when these thresholds are reached prematurely, monitoring must be intensified to assess the survival and success potential of the third generation (*G*_3_), thereby enhancing early detection frameworks for invasive forest pests [60].

Management of Stand Structure and Structural Diversification

Given the statistically demonstrated “Edge Effect” (Kruskal–Wallis, *p* < 0.001), sustainable management must focus on reducing forest fragmentation and modifying edge dynamics, as microclimatic variables and light environments strongly dictate the intensity of herbivorous insect attacks [61]. To mitigate these impacts, forest management must shift toward structural diversification. We recommend the establishment of “microclimatic buffers”—dense, multi-layered shrub belts composed of non-host species. These buffers serve a dual purpose: they reduce direct solar radiation on the forest edge, keeping the local Aridity Index above the critical threshold of 20, and physically obstruct the wind-driven penetration of the pest into the forest core.

Resilience Recovery and Treatment Optimization

The SPAD degradation data underscore the importance of identifying *Quercus petraea* phenotypes that maintain high photosynthetic efficiency even under intense attack impact. Assisted regeneration programs should prioritize genetic material from specimens that have demonstrated resilience in the most affected areas (the Rășinari forest edges). Furthermore, understanding the windows of ethological vulnerability, such as nymphal aggregation, allows for the optimization of biological treatment timing. Interventions should target the early stages of the G_1_ and G_2_ generations before vertical migration phenomena render the populations difficult to access within the interior of the canopy.

### 4.6. Implications for Forest Management and Resilience

Our findings suggest that forest managers should adopt a proactive monitoring strategy. In Southern Transylvania (Romania), an Aridity Index (I_Ar_) falling below 20 during the June–July period serves as a reliable early-warning indicator for *C. arcuata* outbreaks. Furthermore, the higher LSI observed at the forest edges (0–20 m) suggests that diversifying edge species with non-host trees or creating denser shrub buffers could mitigate the ‘edge effect’ and limit the insect’s penetration into the forest core.

Our management recommendations must be viewed through the lens of oak ecology. *Quercus* spp. are light-demanding species that often fail to regenerate under closed canopies, being replaced by shade-tolerant taxa such as *Tilia* or *Acer*. As suggested by recent palaeoecological evidence, primeval European landscapes were likely herbivory-driven woodland–grassland mosaics [62] rather than dense, continuous forests. Consequently, maintaining sparse, ‘savanna-like’ stand structures may be essential for oak regeneration, even if such open structures potentially influence the dispersal dynamics of *C. arcuata*.

## 5. Conclusions

The present study provides an integrated perspective on the mechanisms facilitating the invasive expansion of *Corythucha arcuata* within the oak ecosystems of Southern Transylvania. The multidisciplinary analysis conducted between 2024 and 2025 leads to the following fundamental conclusions.

Aridity-Induced Vulnerability: A critical threshold for the De Martonne Aridity Index (I_Ar_ = 20) was identified. Below this value, the physiological resilience of *Quercus petraea* appears to decline drastically. The strong negative correlation (r_s_ = −0.84) between water deficit and SPAD values suggests that drought may function not merely as a parallel stressor, but as a catalyst that potentially primes the host substrate for pest infestation.

Host–Pest Biochemical Synergy: Biochemical results are consistent with a metabolic reconfiguration of leaves subjected to thermo-hydric stress, marked by a 58.8% increase in soluble sugars. This accumulation supports the hypothesis of a “metabolic bait” (phagostimulant) mechanism, which is consistent with the population explosion of the G_2_ generation and the critical densities observed at forest edges.

Habitat Dynamics and the “Edge Effect”: Statistical analysis (*p* < 0.001) is consistent with the view that forest fragmentation and the direct exposure of edges create “heat island” microclimates. These conditions are associated with accelerated nymphal development and a complex voltinism pattern. Our data identifies two complete generations and a partial third generation (G_3_), with the latter being potentially limited by early frosts, as indicated by a survival rate of <12%.

Ethological Resilience Adaptations: The study highlights the behavioral thermoregulation capacity of *C. arcuata*. Vertical migration into the interior of the canopy when leaf temperatures exceed the identified 32 °C threshold, coupled with nymphal aggregation on the abaxial leaf surface, are supported as key ethological strategies. These behaviors are consistent with the 92% survival rate recorded for the summer generation despite extreme heatwaves.

Implications for Sustainable Forest Management: We propose the implementation of early warning systems based on the monitoring of I_Ar_ indices and leaf thermal thresholds. Sustainable management should prioritize the reduction of forest fragmentation through the creation of buffer zones (gradual ecotones) and the selection of oak phenotypes that demonstrate photosynthetic stability under biotic impact.

Ultimately, the decline of oak stands within the Rășinari Forest District supports a model wherein the infestation transcends a simple pest outbreak. It is consistent with a ‘perfect storm’ scenario where cumulative abiotic stress potentially compromises the host’s physiological defenses, transforming an invasive pest from a secondary stressor into a significant driver of ecosystem mortality.

## Figures and Tables

**Table 1:** Multiple comparisons post hoc test results.

Habitat Comparison	Rank Difference	Z-Score	*p*-Value (Adjusted)
Forest Edge vs. Forest Interior	88.2	4.15	<0.001
Forest Edge vs. Ecotone	43.7	2.51	0.012
Ecotone vs. Forest Interior	44.5	2.54	0.011

## Data Availability

The data presented in this study are available on request from the corresponding authors.
